# Supplementary material for: Low educational level increases functional disability risk subsequent to heart failure in Japan: On behalf of the Iwate KENCO study group
Source: PLoS One. 2021 Jun 8;16(6):e0253017. doi: 10.1371/journal.pone.0253017 (PMC8186788; doi:10.1371/journal.pone.0253017)
Supplement: S1 Table — (DOCX) [file pone.0253017.s001.docx]

**S1 Table. Odds ratios (95% confidence interval) for the categories of no long-term care insurance after heart failure and long-term care insurance after heart failure excluding subjects with sub-clinical.**

|  | **No LTCI after HF** | | |  | **LTCI after HF** | | |
| --- | --- | --- | --- | --- | --- | --- | --- |
|  | **Model 1** | **Model 2** | **Model 3** |  | **Model 1** | **Model 2** | **Model 3** |
|  | **OR (95% CI)** | **OR (95% CI)** | **OR (95% CI)** |  | **OR (95% CI)** | **OR (95% CI)** | **OR (95% CI)** |
| **Current smoker** | 1.03 (0.38– 2.82) | 1.02 (0.34– 3.04) | 1.05 (0.35– 3.15) |  | 0.58 (0.15– 2.33) | 0.67 (0.15– 2.88) | 0.51 (0.11– 2.27) |
| **Regular drinker** | 0.65 (0.24– 1.76) | 0.69 (0.24– 1.99) | 0.77 (0.26– 2.27) |  | 2.47 (0.85– 7.16) | 2.22 (0.75– 6.63) | 2.66 (0.78– 9.08) |
| **Hypertension** |  | 1.77 (0.82– 3.81) | 1.78 (0.83– 3.84) |  |  | 1.37 (0.59– 3.21) | 1.46 (0.54– 3.93) |
| **Diabetes mellitus** |  | 1.15 (0.26– 5.04) | 1.13 (0.25– 5.06) |  |  | 0.97 (0.17– 5.57) | 0.87 (0.13– 5.82) |
| **Dyslipidemia** |  | 0.54 (0.24– 1.22) | 0.53 (0.23– 1.20) |  |  | 0.43 (0.15– 1.22) | 0.33 (0.10– 1.04) |
| **Unmarried status** |  |  | 1.12 (0.47– 2.65) |  |  |  | 1.96 (0.63– 6.04) |
| **Lower educational level** |  |  | 1.07 (0.46– 2.49) |  |  |  | 3.75 (1.37–10.30) |
| **Unemployed status** |  |  | 1.54 (0.75– 3.20) |  |  |  | 0.53 (0.15– 1.80) |

LTCI, long-term care insurance; HF, heart failure; OR, odds ratio; CI, confidence interval.
