# Supplementary material for: Low educational level increases functional disability risk subsequent to heart failure in Japan: On behalf of the Iwate KENCO study group
Source: PLoS One. 2021 Jun 8;16(6):e0253017. doi: 10.1371/journal.pone.0253017 (PMC8186788; doi:10.1371/journal.pone.0253017)
Supplement: S2 Table — (PDF) [file pone.0253017.s002.pdf]

S2 Table. Data in the present study.

age	BMI	sbp	dbp	TC	HDLC	nonHDLC	HB	Hba1c_N	egfr_epi	sex	age_n	TBC_SITU_c	ALC_Reg	t1htt_in	dm_ni	t1lipid_o	Q_MARR_s	Q_school_t	Q05_s	Bunrui	y_moku2	y_moku	filter_Apr0220x
76	29.7	139	86	132	48	84	9.8	6	75	1	3	1	0	0	1	1	2.00	2	0	1	1	1	0
66	25.2	149	84	219	39	180	13.4	5	76	1	1	1	1	0	1	0	2.00	2	0	1	1	1	0
67	21.6	145	93	220	49	171	14.9	6	79	1	1	1	0	0	1	0	2.00	1	1	1	1	1	0
65	19.3	139	71	149	81	68	13.8	5	64	1	1	1	0	1	0	1	2.00	2	0	1	1	1	0
83	20.1	126	79	240	103	137	11.7	6	56	2	4	1	1	0	1	0	2.00	1	1	1	1	1	0
69	23.3	146	82	176	67	109	13.9	6	61	2	1	1	1	0	0	1	1.00	2	1	1	1	1	0
78	27.5	129	70	188	46	142	12.3	6	73	1	3	1	1	0	1	1	2.00	2	0	1	1	1	0
74	20.0	151	85	298	62	236	14.9	6	68	1	2	1	1	0	1	0	2.00	2	0	1	1	1	0
70	29.1	175	87	206	47	159	14.3	6	74	1	2	0	1	0	1	1	2.00	2	0	1	1	1	0
65	19.2	109	64	146	48	98	13.1	7	48	2	1	1	1	1	0	1	2.00	2	0	1	1	1	0
72	22.4	151	76	192	81	111	12.1	5	79	2	2	1	1	0	1	1	2.00	1	1	1	1	1	1
65	23.9	152	90	205	71	134	14.2	5	78	2	1	1	1	0	1	1	1.00	2	0	1	1	1	1
72	26.0	145	83	193	40	153	13.0	9	82	1	2	0	1	0	0	1	1.00	2	0	1	1	1	1
72	22.3	135	49	166	66	100	15.1	5	77	1	2	0	0	0	1	1	2.00	2	0	1	1	1	1
77	16.1	187	102	212	48	164	13.6	6	50	2	3	1	1	0	1	1	1.00	1	0	1	1	1	1
75	18.0	118	68	204	93	111	13.9	5	75	1	3	1	0	1	1	1	2.00	2	0	1	1	1	1
70	19.9	148	87	158	65	93	13.8	5	55	1	2	1	1	0	1	1	2.00	2	1	1	1	1	1
72	26.8	161	88	263	85	178	15.3	5	71	2	2	1	1	0	1	0	2.00	2	0	1	1	1	1
75	23.1	141	80	229	80	149	13.6	5	73	2	3	1	1	0	1	0	1.00	1	0	1	1	1	1
70	27.2	125	74	179	54	125	12.9	6	61	2	2	1	1	0	1	1	1.00	2	0	1	1	1	1
74	20.1	101	68	147	40	107	12.7	6	68	1	2	0	1	1	1	1	1.00	2	0	1	1	1	1
70	26.8	159	84	196	47	149	14.4	7	61	2	2	1	1	0	0	1	2.00	2	0	1	1	1	1
71	18.8	126	52	155	54	101	14.2	6	82	1	2	0	1	1	1	1	2.00	2	1	1	1	1	1
77	26.4	157	81	173	48	125	14.0	5	68	2	3	1	1	0	1	1	2.00	1	1	1	1	1	1
70	23.4	108	63	154	29	125	12.8	6	45	1	2	1	1	0	1	0	2.00	2	1	1	1	1	1
74	27.6	130	74	218	62	156	15.2	5	59	2	2	1	1	0	1	1	2.00	2	0	1	1	1	1
73	23.1	147	78	167	43	124	13.9	6	76	1	2	1	0	0	1	1	2.00	2	1	1	1	1	1
72	17.1	135	76	202	82	120	12.0	6	74	2	2	1	1	1	1	1	2.00	2	0	1	1	1	1
72	21.7	136	79	235	41	194	15.6	5	73	1	2	1	1	0	1	0	2.00	2	0	1	1	1	1
68	25.1	121	74	185	72	113	15.6	6	63	1	1	0	0	0	1	1	2.00	2	0	1	1	1	1
71	25.6	137	66	181	45	136	15.1	5	61	1	2	1	1	1	1	1	2.00	1	0	1	1	1	1
70	30.1	121	70	153	49	104	13.6	5	78	1	2	1	1	0	1	1	2.00	2	1	1	1	1	1
67	22.1	126	72	150	58	92	12.5	6	77	2	1	1	1	1	1	1	2.00	2	0	1	1	1	1
71	26.7	160	84	260	90	170	13.6	5	79	2	2	1	1	0	1	0	1.00	2	0	1	1	1	1
75	21.6	122	67	150	52	98	14.3	6	59	2	3	1	1	1	1	1	1.00	2	0	1	1	1	1
76	21.9	149	87	146	69	77	13.6	5	72	2	3	1	1	0	1	1	1.00	1	0	1	1	1	1
71	22.4	111	62	175	70	105	12.0	5	79	2	2	1	1	1	1	1	2.00	1	1	1	1	1	1
66	27.3	112	77	175	49	126	15.3	6	72	1	1	1	0	1	1	1	2.00	2	0	1	1	1	1
76	24.3	164	91	239	56	183	15.7	7	71	1	3	0	1	0	0	0	2.00	2	0	1	1	1	1
82	22.2	105	66	185	82	103	15.3	5	68	1	4	1	0	1	1	1	2.00	1	1	1	1	1	1
75	23.3	157	75	186	71	115	12.1	5	69	2	3	1	1	0	1	1	2.00	2	0	1	1	1	1
71	23.5	115	65	191	80	111	10.3	5	75	2	2	1	1	1	1	1	1.00	2	1	1	1	1	1
67	24.4	141	87	175	67	108	14.0	5	75	1	1	1	0	0	1	1	2.00	2	0	1	1	1	1
77	22.8	138	80	187	75	112	14.5	5	70	1	3	1	1	1	1	1	2.00	1	0	1	1	1	1
78	28.4	136	86	187	31	156	14.7	5	52	1	3	1	1	0	1	0	2.00	2	0	1	1	1	1
79	20.4	130	67	158	38	120	13.8	6	73	1	3	0	1	1	1	0	1.00	2	1	1	1	1	1
76	20.2	145	86	179	65	114	14.3	6	75	1	3	1	1	0	1	1	2.00	2	1	1	1	1	1

70	21.0	192	106	213	99	114	12.3	6	72	2	2	1	1	0	1	1	2.00	1	0	1	1	1	1
79	21.4	134	71	229	60	169	13.9	5	58	1	3	1	1	1	1	0	2.00	2	0	1	1	1	1
69	24.1	103	64	219	41	178	15.4	5	71	1	1	1	1	1	1	1	2.00	2	0	1	1	1	1
69	37.4	150	89	228	56	172	16.0	5	81	2	1	1	1	0	1	0	2.00	1	1	1	1	1	1
70	24.3	110	63	214	51	163	12.9	6	72	2	2	1	1	1	1	1	1.00	1	0	1	1	1	1
71	20.1	120	72	189	65	124	12.5	5	75	2	2	1	1	1	1	1	2.00	1	1	2	1	1	0
71	28.2	155	79	231	70	161	12.9	5	75	2	2	1	1	0	1	0	1.00	1	0	2	1	1	0
77	25.4	142	79	178	63	115	15.3	5	67	1	3	1	0	0	1	1	1.00	2	0	2	1	1	0
73	18.4	88	55	188	55	133	12.2	6	72	1	2	1	0	1	1	1	2.00	2	1	2	1	1	0
68	22.6	153	82	240	59	181	12.2	5	73	2	1	1	1	0	1	0	2.00	1	0	2	1	1	0
66	29.5	116	70	251	53	198	13.7	6	74	2	1	1	1	1	1	0	1.00	2	0	2	1	1	0
76	21.3	152	70	175	52	123	13.6	6	51	2	3	1	1	0	1	1	1.00	2	0	2	1	1	0
75	25.8	140	85	255	50	205	14.1	6	71	1	3	0	0	0	1	0	2.00	1	0	2	1	1	0
76	23.8	154	87	243	58	185	15.0	6	75	1	3	1	1	0	1	0	2.00	2	1	2	1	1	0
72	22.8	131	84	160	76	84	14.2	6	73	1	2	1	0	1	1	1	2.00	1	0	2	1	1	0
66	24.9	137	59	181	51	130	13.4	5	82	2	1	1	0	1	1	1	2.00	1	1	2	1	1	0
74	27.9	155	84	180	44	136	11.9	6	76	1	2	1	1	0	1	1	2.00	1	0	2	1	1	1
69	20.7	172	99	169	58	111	14.7	5	78	1	1	1	0	0	1	1	2.00	2	1	2	1	1	1
71	21.2	157	80	162	58	104	14.0	5	71	2	2	1	1	0	1	1	2.00	1	0	2	1	1	1
70	27.2	127	79	182	74	108	12.8	6	80	2	2	1	1	1	1	1	1.00	1	1	2	1	1	1
79	24.7	136	83	204	43	161	13.7	5	67	2	3	1	1	1	1	1	2.00	2	0	2	1	1	1
78	29.4	145	76	202	44	158	13.5	7	71	2	3	1	1	0	0	1	1.00	1	0	2	1	1	1
77	24.5	158	76	229	51	178	13.5	6	72	2	3	1	1	0	1	0	1.00	1	0	2	1	1	1
72	25.6	140	94	201	36	165	15.2	5	73	1	2	1	0	0	1	0	2.00	2	1	2	1	1	1
71	16.0	104	66	156	43	113	11.6	5	75	2	2	1	1	1	1	1	2.00	1	0	2	1	1	1
74	22.0	122	72	196	40	156	15.0	6	68	1	2	1	1	1	1	1	2.00	1	1	2	1	1	1
76	17.9	115	57	193	46	147	10.1	5	23	2	3	1	1	1	1	1	1.00	2	0	2	1	1	1
70	21.5	135	77	146	66	80	12.6	5	78	1	2	1	1	1	1	1	2.00	1	1	2	1	1	1
87	30.0	126	67	142	49	93	13.2	5	54	2	4	1	1	1	1	1	2.00	1	0	2	1	1	1
73	21.0	102	59	164	75	89	13.2	5	76	1	2	0	0	1	1	1	1.00	2	0	2	1	1	1
75	20.7	180	87	208	56	152	13.3	6	71	1	3	0	1	0	1	1	1.00	1	0	2	1	1	1
69	26.8	146	76	242	53	189	14.5	5	81	2	1	1	1	0	1	0	2.00	1	1	2	1	1	1
71	21.0	142	68	211	84	127	12.7	5	77	1	2	1	0	0	1	1	1.00	2	1	2	1	1	1
69	21.6	134	77	212	67	145	14.4	5	61	2	1	1	1	1	1	1	2.00	2	0	2	1	1	1
75	27.7	136	70	189	51	138	12.7	5	59	2	3	1	1	0	1	1	1.00	2	0	2	1	1	1
80	22.4	151	82	148	52	96	12.6	5	69	1	4	1	1	0	1	1	2.00	2	0	2	1	1	1
81	19.6	127	78	146	61	85	12.2	6	68	1	4	1	0	1	1	1	2.00	1	0	2	1	1	1
71	22.5	118	73	158	38	120	16.9	6	77	1	2	0	0	1	0	0	2.00	2	1	2	1	1	1
84	18.1	154	78	168	49	119	12.6	6	70	1	4	1	0	0	1	1	2.00	2	0	2	1	1	1
77	24.3	164	84	198	91	107	13.8	6	72	2	3	1	1	0	1	1	1.00	1	0	2	1	1	1
78	25.6	112	63	139	46	93	16.6	5	58	1	3	1	1	1	1	1	2.00	2	0	2	1	1	1
76	27.2	122	70	182	61	121	12.2	6	77	2	3	1	1	0	1	1	1.00	2	0	2	1	1	1
74	33.8	173	103	129	32	97	14.4	6	72	1	2	1	1	0	1	0	2.00	2	1	2	1	1	1
71	28.5	154	78	181	61	120	15.8	5	73	1	2	1	0	0	1	1	1.00	2	1	2	1	1	1
80	27.4	132	72	146	38	108	12.7	6	67	2	4	1	1	0	1	0	1.00	1	0	2	1	1	1
82	28.3	143	68	204	42	162	13.6	6	64	1	4	1	1	0	1	1	2.00	2	0	2	1	1	1
76	21.3	168	79	210	97	113	14.0	5	75	1	3	1	0	0	1	1	2.00	1	1	2	1	1	1
80	22.7	91	54	181	46	135	11.3	6	70	2	4	1	1	1	1	1	1.00	1	0	2	1	1	1
79	23.7	118	62	215	48	167	10.7	6	67	2	3	1	1	0	1	1	1.00	1	1	2	1	1	1
76	22.4	138	76	196	50	146	13.5	7	59	1	3	1	1	0	0	1	1.00	2	0	1	2	0	0
76	21.3	168	79	210	97	113	14.0	5	75	1	3	1	0	0	1	1	2.00	1	1	1	2	0	0

76	28.2	141	75	192	57	135	13.6	6	71	1	3	1	1	0	1	1	2.00	2	0	1	2	0	0
66	23.0	104	64	218	69	149	14.2	5	72	1	1	0	1	1	1	1	2.00	2	1	1	2	0	0
66	25.7	159	88	218	44	174	16.3	5	76	1	1	1	0	0	1	1	2.00	2	1	1	2	0	0
66	23.4	159	88	245	54	191	15.2	6	76	1	1	0	0	0	1	0	2.00	1	0	1	2	0	0
67	24.8	99	65	202	62	140	14.3	5	72	1	1	1	1	1	1	1	2.00	2	1	1	2	0	0
67	20.6	123	72	205	62	143	17.9	5	75	1	1	0	0	1	1	1	2.00	1	1	1	2	0	0
67	23.4	104	63	148	46	102	14.8	5	75	1	1	1	1	1	1	1	2.00	2	1	1	2	0	0
65	24.8	133	91	198	42	156	15.4	5	76	1	1	0	0	0	1	1	2.00	2	1	1	2	0	0
65	18.7	125	68	174	57	117	13.0	5	73	1	1	0	1	1	1	1	2.00	2	0	1	2	0	0
65	18.0	115	70	182	61	121	14.5	6	81	1	1	1	1	1	1	1	2.00	2	0	1	2	0	0
83	18.7	112	68	204	60	144	10.0	5	65	2	4	1	1	1	1	1	1.00	1	0	1	2	0	0
83	24.2	151	86	178	55	123	14.2	5	56	2	4	1	1	0	1	1	2.00	1	0	1	2	0	0
83	21.8	137	63	185	77	108	11.2	6	65	2	4	1	1	1	1	1	1.00	1	0	1	2	0	0
69	23.6	109	69	176	64	112	12.3	5	81	2	1	1	1	1	1	1	2.00	2	1	1	2	0	0
69	21.0	99	62	171	55	116	11.1	5	81	2	1	1	1	1	1	1	2.00	1	0	1	2	0	0
69	26.3	155	83	226	77	149	12.2	6	81	2	1	1	1	0	1	0	2.00	1	1	1	2	0	0
78	23.6	172	88	245	56	189	14.7	6	58	1	3	1	0	0	1	0	2.00	2	1	1	2	0	0
78	21.0	149	83	200	58	142	11.7	5	70	1	3	1	1	0	1	1	2.00	2	1	1	2	0	0
78	21.5	91	55	220	73	147	11.9	5	70	1	3	1	0	1	1	0	2.00	2	0	1	2	0	0
74	23.4	139	73	217	40	177	13.6	6	72	1	2	0	1	1	1	1	2.00	2	1	1	2	0	0
74	25.3	149	91	196	49	147	14.3	6	53	1	2	1	1	0	1	1	2.00	2	0	1	2	0	0
74	27.6	159	98	211	44	167	16.0	6	68	1	2	1	1	0	1	1	2.00	2	0	1	2	0	0
70	21.5	148	89	195	55	140	15.3	5	78	1	2	0	1	0	1	1	2.00	2	0	1	2	0	0
70	21.7	155	78	287	53	234	14.2	5	78	1	2	1	1	0	1	0	2.00	2	0	1	2	0	0
70	25.6	123	72	164	63	101	14.4	7	78	1	2	1	0	1	0	1	2.00	2	0	1	2	0	0
65	23.1	138	84	235	70	165	12.7	6	74	2	1	1	1	0	1	0	2.00	2	0	1	2	0	0
65	26.0	135	77	189	77	112	10.7	5	78	2	1	1	1	1	1	0	2.00	2	1	1	2	0	0
65	23.0	115	67	187	58	129	14.0	5	83	2	1	1	0	1	1	1	2.00	2	1	1	2	0	0
72	24.4	116	72	208	33	175	12.3	5	74	2	2	1	1	1	1	0	2.00	2	1	1	2	0	1
72	24.5	145	85	186	71	115	11.8	6	71	2	2	1	1	0	1	1	2.00	1	0	1	2	0	1
72	23.2	111	62	125	46	79	12.9	6	74	2	2	1	1	1	1	1	1.00	2	0	1	2	0	1
65	25.7	129	88	220	72	148	12.0	5	74	2	1	1	1	1	1	0	1.00	2	0	1	2	0	1
65	30.1	117	72	217	62	155	14.7	6	78	2	1	1	0	1	1	1	2.00	2	1	1	2	0	1
65	26.6	155	86	196	66	130	12.7	5	78	2	1	1	1	0	1	1	2.00	2	0	1	2	0	1
72	25.0	117	64	173	37	136	15.1	5	61	1	2	1	0	1	1	0	1.00	2	0	1	2	0	1
72	23.6	124	75	179	66	113	14.1	5	73	1	2	1	1	1	1	1	2.00	2	0	1	2	0	1
72	29.1	157	96	237	67	170	14.4	6	73	1	2	0	1	0	1	0	2.00	2	1	1	2	0	1
72	19.4	101	60	189	58	131	13.2	5	73	1	2	1	0	1	1	1	2.00	1	0	1	2	0	1
72	23.0	126	76	215	91	124	15.3	6	69	1	2	0	0	1	1	1	2.00	1	1	1	2	0	1
72	23.0	95	63	199	50	149	14.3	6	69	1	2	0	1	1	1	0	2.00	2	0	1	2	0	1
77	27.2	136	65	164	49	115	15.0	5	72	2	3	1	1	0	1	1	2.00	1	0	1	2	0	1
77	22.7	121	66	238	39	199	12.9	5	68	2	3	1	1	1	1	0	1.00	1	1	1	2	0	1
77	26.9	112	64	208	99	109	13.7	7	72	2	3	1	1	1	0	0	2.00	2	0	1	2	0	1
75	22.1	175	101	144	41	103	13.9	5	68	1	3	0	1	0	1	1	1.00	2	0	1	2	0	1
75	22.8	156	72	218	57	161	16.2	8	68	1	3	0	0	0	0	1	2.00	2	1	1	2	0	1
75	26.1	153	84	202	55	147	16.0	6	68	1	3	1	1	0	1	1	2.00	2	0	1	2	0	1
70	25.5	129	66	213	39	174	15.1	6	74	1	2	0	1	0	1	0	2.00	2	0	1	2	0	1
70	25.4	164	105	155	40	115	14.9	6	83	1	2	0	1	0	1	1	2.00	2	1	1	2	0	1
70	19.8	121	81	211	69	142	15.2	5	83	1	2	1	0	1	1	1	2.00	1	0	1	2	0	1
72	24.0	117	69	165	40	125	12.4	5	71	2	2	1	1	0	1	1	1.00	2	0	1	2	0	1
72	20.7	117	73	178	53	125	12.7	5	60	2	2	1	1	0	1	1	2.00	2	0	1	2	0	1

72	26.7	143	81	205	47	158	13.8	6	79	2	2	1	1	0	1	1	1.00	1	1	1	2	0	1
75	25.5	132	69	172	49	123	13.4	5	59	2	3	1	1	1	1	1	1.00	2	0	1	2	0	1
75	22.6	146	76	228	45	183	12.9	8	51	2	3	1	1	0	0	0	1.00	2	0	1	2	0	1
75	22.0	134	75	196	58	138	11.2	6	73	2	3	1	1	1	1	1	2.00	2	0	1	2	0	1
70	22.9	126	69	210	73	137	12.7	5	72	2	2	1	1	0	1	0	2.00	1	1	1	2	0	1
70	19.7	113	71	166	49	117	11.5	5	75	2	2	1	1	1	1	1	2.00	1	1	1	2	0	1
70	22.3	134	78	225	68	157	13.8	6	75	2	2	1	1	1	1	0	2.00	2	0	1	2	0	1
74	22.4	147	91	192	45	147	14.9	6	76	1	2	1	1	0	1	1	2.00	2	1	1	2	0	1
74	29.3	134	85	167	58	109	14.3	5	76	1	2	1	0	1	1	1	2.00	2	0	1	2	0	1
74	23.3	160	91	229	73	156	14.4	6	72	1	2	1	0	0	1	0	2.00	1	0	1	2	0	1
70	24.8	132	89	223	49	174	12.4	6	75	2	2	1	1	1	1	0	1.00	1	1	1	2	0	1
70	30.8	154	89	225	47	178	14.4	6	72	2	2	1	1	0	1	0	2.00	2	0	1	2	0	1
70	22.3	134	78	225	68	157	13.8	6	75	2	2	1	1	1	1	0	2.00	2	0	1	2	0	1
71	24.6	125	72	191	50	141	14.1	6	73	1	2	1	1	1	1	1	2.00	2	0	1	2	0	1
71	22.7	124	67	249	46	203	14.9	7	70	1	2	0	1	1	0	0	2.00	2	1	1	2	0	1
71	24.3	125	83	186	52	134	17.7	6	61	1	2	0	0	1	1	1	2.00	2	0	1	2	0	1
77	22.8	113	59	223	40	183	12.2	6	72	2	3	1	1	0	1	0	2.00	1	0	1	2	0	1
77	21.1	144	80	193	46	147	13.4	5	76	2	3	1	1	0	1	1	1.00	2	0	1	2	0	1
77	24.6	147	90	162	46	116	11.4	6	68	2	3	1	1	0	1	1	2.00	1	0	1	2	0	1
70	22.9	118	65	170	67	103	15.3	8	78	1	2	1	1	1	0	1	2.00	1	0	1	2	0	1
70	20.6	110	61	234	55	179	15.1	6	70	1	2	0	0	1	1	0	2.00	2	1	1	2	0	1
70	25.1	128	81	182	42	140	16.1	6	78	1	2	1	1	1	1	1	2.00	2	0	1	2	0	1
77	24.3	164	84	198	91	107	13.8	6	72	2	3	1	1	0	1	1	1.00	1	0	1	2	0	1
74	28.8	151	78	219	57	162	12.2	6	73	2	2	1	1	0	1	1	2.00	1	0	1	2	0	1
74	24.1	86	46	241	83	158	12.1	5	73	2	2	1	1	1	1	0	2.00	2	0	1	2	0	1
73	24.7	131	74	156	42	114	14.6	5	72	1	2	1	1	1	1	1	1.00	1	0	1	2	0	1
73	21.4	122	83	167	38	129	13.5	6	72	1	2	0	0	1	1	0	2.00	2	0	1	2	0	1
73	24.5	131	68	211	64	147	13.4	5	69	1	2	1	1	0	1	1	2.00	1	1	1	2	0	1
72	25.4	107	63	221	82	139	12.7	5	60	2	2	1	1	1	1	0	2.00	1	0	1	2	0	1
72	28.4	162	81	261	38	223	12.6	5	60	2	2	1	1	0	1	0	1.00	2	1	1	2	0	1
72	24.1	116	73	207	32	175	12.7	6	74	2	2	1	1	1	1	0	1.00	2	1	1	2	0	1
72	21.6	144	79	201	35	166	15.1	4	61	1	2	1	0	0	1	0	1.00	2	0	1	2	0	1
72	23.8	154	82	224	37	187	14.6	6	77	1	2	0	1	0	1	0	2.00	2	0	1	2	0	1
72	25.3	143	76	188	67	121	13.1	6	73	1	2	1	0	0	1	1	2.00	1	1	1	2	0	1
68	21.0	112	81	204	50	154	14.4	6	75	1	1	1	1	1	1	1	2.00	2	0	1	2	0	1
68	21.9	132	95	187	53	134	12.8	5	71	1	1	1	0	0	1	1	2.00	2	1	1	2	0	1
68	23.6	124	67	220	63	157	14.5	6	79	1	1	1	0	0	1	0	2.00	2	0	1	2	0	1
71	22.5	103	68	177	61	116	13.5	5	73	1	2	1	0	1	1	1	2.00	2	1	1	2	0	1
71	20.3	113	58	197	75	122	12.8	7	70	1	2	0	0	0	0	1	2.00	2	1	1	2	0	1
71	24.2	124	71	152	55	97	14.2	5	82	1	2	1	1	1	1	1	2.00	2	1	1	2	0	1
70	25.8	106	71	195	36	159	14.9	5	74	1	2	1	1	1	1	0	2.00	2	0	1	2	0	1
70	22.1	116	71	148	43	105	15.0	4	74	1	2	1	0	1	1	1	2.00	1	0	1	2	0	1
70	22.3	119	70	177	47	130	13.6	5	74	1	2	1	1	0	1	1	2.00	2	1	1	2	0	1
67	24.0	118	66	225	56	169	14.5	6	73	2	1	1	1	0	1	0	1.00	2	0	1	2	0	1
67	23.2	130	72	269	93	176	13.1	5	73	2	1	1	1	1	1	0	2.00	1	1	1	2	0	1
67	26.5	108	74	217	62	155	12.9	6	77	2	1	1	1	1	1	1	2.00	2	1	1	2	0	1
71	19.6	132	72	246	95	151	12.8	5	71	2	2	1	1	1	1	0	2.00	2	0	1	2	0	1
71	27.3	118	74	202	61	141	15.2	6	79	2	2	1	0	1	1	1	1.00	2	1	1	2	0	1
71	27.7	142	79	181	35	146	13.7	6	71	2	2	1	1	0	1	0	1.00	2	0	1	2	0	1
75	24.0	92	47	162	46	116	12.7	5	83	2	3	1	1	1	1	1	1.00	1	1	1	2	0	1
75	25.4	152	84	231	41	190	12.7	6	59	2	3	1	1	0	1	0	1.00	1	0	1	2	0	1

75	23.4	137	80	213	57	156	13.6	5	77	2	3	1	1	1	1	0	2.00	1	1	1	2	0	1
76	22.2	139	78	242	74	168	15.1	6	58	2	3	1	1	1	1	0	1.00	2	0	1	2	0	1
76	23.1	130	68	130	40	90	10.7	5	72	2	3	1	1	1	1	1	2.00	2	0	1	2	0	1
76	19.1	161	82	215	57	158	12.2	5	72	2	3	1	1	0	1	1	2.00	1	1	1	2	0	1
71	27.8	142	77	197	58	139	14.4	6	75	2	2	1	1	0	1	1	2.00	2	0	1	2	0	1
71	24.2	121	69	161	65	96	13.9	6	75	2	2	1	1	0	1	1	2.00	2	0	1	2	0	1
71	24.5	153	89	208	55	153	14.1	6	75	2	2	1	1	0	1	1	2.00	2	1	1	2	0	1
66	25.3	130	72	184	38	146	14.7	6	80	1	1	0	0	1	1	0	2.00	2	1	1	2	0	1
66	23.0	153	91	182	78	104	14.3	5	63	1	1	0	0	0	1	1	2.00	2	0	1	2	0	1
66	24.6	122	66	185	58	127	15.3	6	80	1	1	1	1	1	1	1	2.00	2	1	1	2	0	1
76	32.6	143	93	152	54	98	14.2	5	47	1	3	1	1	0	1	1	2.00	2	0	1	2	0	1
76	26.2	107	58	245	48	197	14.0	6	71	1	3	1	1	1	1	0	2.00	2	1	1	2	0	1
76	22.4	172	89	253	51	202	16.5	6	59	1	3	1	1	0	1	0	2.00	1	0	1	2	0	1
82	21.4	134	72	185	58	127	13.6	5	68	1	4	0	0	1	1	1	2.00	2	1	1	2	0	1
82	24.7	112	65	140	44	96	11.5	6	51	1	4	1	1	1	1	1	1.00	2	0	1	2	0	1
82	28.3	143	68	204	42	162	13.6	6	64	1	4	1	1	0	1	1	2.00	2	0	1	2	0	1
75	20.9	105	60	222	56	166	10.0	5	77	2	3	1	1	1	1	0	2.00	1	0	1	2	0	1
75	18.5	99	64	222	71	151	13.6	6	77	2	3	1	1	1	1	0	1.00	2	1	1	2	0	1
75	25.2	146	88	193	58	135	13.2	5	73	2	3	1	1	0	1	1	1.00	2	1	1	2	0	1
71	23.9	170	84	229	71	158	14.6	5	75	2	2	1	1	0	1	0	2.00	1	0	1	2	0	1
71	21.0	151	85	171	43	128	13.9	5	75	2	2	1	1	0	1	1	2.00	2	0	1	2	0	1
71	21.4	143	76	189	53	136	12.9	5	75	2	2	1	1	0	1	1	1.00	1	1	1	2	0	1
67	25.6	121	67	177	52	125	13.6	5	63	1	1	1	1	0	1	1	2.00	2	0	1	2	0	1
67	23.9	120	75	249	39	210	16.3	5	79	1	1	1	0	0	1	0	2.00	2	1	1	2	0	1
67	24.1	120	78	218	74	144	15.6	5	75	1	1	0	0	0	1	1	2.00	2	1	1	2	0	1
77	22.7	138	74	144	49	95	15.5	8	70	1	3	0	1	1	0	1	2.00	1	0	1	2	0	1
77	28.0	122	68	165	45	120	14.5	6	67	1	3	0	0	1	1	1	2.00	2	1	1	2	0	1
77	20.9	158	93	216	62	154	15.1	5	52	1	3	1	1	0	1	1	2.00	2	0	1	2	0	1
78	21.7	157	84	180	54	126	13.5	5	73	1	3	1	1	0	1	1	2.00	2	1	1	2	0	1
78	27.9	109	62	187	56	131	12.2	5	70	1	3	1	1	0	1	1	2.00	1	1	1	2	0	1
78	20.2	117	65	214	64	150	12.8	5	58	1	3	1	1	1	1	1	2.00	2	1	1	2	0	1
79	22.8	119	68	157	35	122	15.3	5	66	1	3	1	0	1	1	0	2.00	2	1	1	2	0	1
79	22.9	125	73	165	68	97	14.0	5	73	1	3	1	1	1	1	1	2.00	2	1	1	2	0	1
79	25.9	120	74	151	39	112	13.2	5	69	1	3	1	1	0	1	0	2.00	2	0	1	2	0	1
76	20.4	129	68	185	73	112	12.9	5	67	1	3	1	1	1	1	1	2.00	2	0	1	2	0	1
76	24.8	164	84	176	67	109	14.1	5	71	1	3	1	1	0	1	1	2.00	2	1	1	2	0	1
76	19.2	117	73	186	70	116	12.8	5	71	1	3	1	1	1	1	1	2.00	2	0	1	2	0	1
70	27.3	133	86	197	41	156	12.9	6	80	2	2	1	1	0	1	1	1.00	2	1	1	2	0	1
70	21.7	124	86	212	85	127	12.9	5	80	2	2	1	1	0	1	1	1.00	1	1	1	2	0	1
70	19.4	119	76	235	66	169	13.6	6	61	2	2	1	1	1	1	0	1.00	2	0	1	2	0	1
69	19.8	106	66	208	62	146	14.4	5	62	1	1	1	1	1	1	1	2.00	2	0	1	2	0	1
69	26.6	102	59	184	56	128	15.5	6	78	1	1	1	1	1	1	1	2.00	2	1	1	2	0	1
69	23.4	133	74	192	60	132	13.6	5	74	1	1	0	1	1	1	1	2.00	2	0	1	2	0	1
69	26.2	113	60	182	58	124	15.0	5	78	1	1	0	1	1	1	1	2.00	2	0	1	2	0	1
69	21.1	112	70	152	74	78	13.5	6	71	1	1	1	0	1	1	1	2.00	2	1	1	2	0	1
69	24.0	134	77	217	36	181	13.9	5	62	1	1	1	0	1	1	0	2.00	2	0	1	2	0	1
69	18.8	96	61	208	61	147	12.6	5	81	2	1	1	1	1	1	1	2.00	2	1	1	2	0	1
69	26.3	177	95	216	53	163	11.7	6	76	2	1	1	1	0	1	1	2.00	1	1	1	2	0	1
69	20.1	111	63	183	51	132	12.6	5	76	2	1	1	1	0	1	1	1.00	2	0	1	2	0	1
70	21.5	142	88	181	60	121	12.8	6	72	2	2	1	1	0	1	1	1.00	1	0	1	2	0	1
70	21.4	117	64	244	80	164	12.0	5	75	2	2	1	1	1	1	0	2.00	1	0	1	2	0	1

70	31.3	149	85	218	70	148	12.3	5	75	2	2	1	1	0	1	0	1.00	2	1	1	2	0	1
71	22.4	141	61	185	49	136	13.0	5	79	2	2	1	1	0	1	1	2.00	2	1	2	2	0	0
71	31.4	159	83	192	38	154	15.1	6	79	2	2	1	1	0	1	0	2.00	2	1	2	2	0	0
71	23.2	142	82	148	52	96	12.5	6	75	2	2	1	1	0	1	1	2.00	1	0	2	2	0	0
71	28.8	178	90	191	64	127	12.0	5	79	2	2	1	0	0	1	1	2.00	2	0	2	2	0	0
71	23.9	170	84	229	71	158	14.6	5	75	2	2	1	1	0	1	0	2.00	1	0	2	2	0	0
71	23.4	127	73	243	74	169	10.9	6	60	2	2	1	1	1	1	0	1.00	2	0	2	2	0	0
77	21.9	148	81	264	95	169	14.4	5	79	1	3	1	1	0	1	0	2.00	1	1	2	2	0	0
77	21.0	128	66	154	63	91	12.8	6	59	1	3	1	1	0	1	1	2.00	2	0	2	2	0	0
77	25.5	129	73	226	47	179	12.8	5	70	1	3	1	1	1	1	0	2.00	2	0	2	2	0	0
73	23.1	147	78	167	43	124	13.9	6	76	1	2	1	0	0	1	1	2.00	2	1	2	2	0	0
73	18.7	130	71	148	64	84	13.0	6	76	1	2	1	0	0	0	1	2.00	2	0	2	2	0	0
73	21.2	101	54	205	65	140	13.9	6	87	1	2	0	1	1	1	1	2.00	2	1	2	2	0	0
68	20.8	106	61	219	113	106	12.8	5	76	2	1	1	1	1	1	1	2.00	2	0	2	2	0	0
68	17.7	132	88	262	68	194	14.3	5	73	2	1	1	1	0	1	0	2.00	2	0	2	2	0	0
68	33.3	119	81	206	44	162	13.7	6	73	2	1	1	1	0	1	1	2.00	2	0	2	2	0	0
66	26.1	119	74	238	63	175	13.6	10	82	2	1	1	1	1	0	0	1.00	2	1	2	2	0	0
66	19.3	106	65	167	56	111	12.2	10	74	2	1	1	1	1	0	1	1.00	2	0	2	2	0	0
66	24.1	119	74	212	33	179	12.5	6	74	2	1	1	1	0	1	0	1.00	2	0	2	2	0	0
76	25.8	145	86	154	38	116	11.4	6	77	2	3	1	1	0	1	0	1.00	2	0	2	2	0	0
76	22.2	150	77	210	62	148	12.4	5	72	2	3	1	1	0	1	1	2.00	2	0	2	2	0	0
76	21.4	165	102	219	47	172	11.7	5	69	2	3	1	1	0	1	1	1.00	1	0	2	2	0	0
75	24.0	115	75	215	42	173	15.5	6	68	1	3	1	0	0	1	1	2.00	2	0	2	2	0	0
75	19.1	127	79	222	60	162	14.0	5	68	1	3	0	1	1	1	0	2.00	2	1	2	2	0	0
75	19.6	131	85	166	43	123	13.8	5	68	1	3	1	1	1	1	1	2.00	2	1	2	2	0	0
76	21.3	168	79	210	97	113	14.0	5	75	1	3	1	0	0	1	1	2.00	1	1	2	2	0	0
76	26.1	122	71	202	52	150	15.7	6	67	1	3	1	1	1	1	1	1.00	2	0	2	2	0	0
76	21.4	107	62	177	38	139	13.6	6	67	1	3	1	1	1	1	0	2.00	2	0	2	2	0	0
72	21.9	126	68	216	80	136	15.7	11	69	1	2	1	1	1	0	1	2.00	1	1	2	2	0	0
72	23.4	143	81	177	53	124	16.6	5	73	1	2	0	0	0	1	1	2.00	1	0	2	2	0	0
72	24.3	133	70	163	51	112	15.0	5	77	1	2	1	0	1	1	1	2.00	2	0	2	2	0	0
66	28.4	122	69	185	56	129	11.6	6	74	2	1	1	1	1	1	1	2.00	2	0	2	2	0	0
66	27.3	140	78	197	48	149	13.9	11	77	2	1	1	1	0	0	1	2.00	2	0	2	2	0	0
66	22.1	113	68	215	57	158	12.3	6	77	2	1	1	1	1	1	1	2.00	2	1	2	2	0	0
74	21.7	112	70	173	56	117	13.0	5	72	1	2	0	0	1	1	1	2.00	2	0	2	2	0	1
74	25.0	146	83	205	35	170	15.1	5	48	1	2	1	1	0	1	0	2.00	2	0	2	2	0	1
74	30.7	135	75	216	47	169	14.6	6	72	1	2	1	0	0	1	1	2.00	2	0	2	2	0	1
69	29.3	144	87	215	51	164	14.7	5	71	1	1	1	1	0	1	1	2.00	2	1	2	2	0	1
69	23.9	128	82	185	68	117	14.0	5	74	1	1	1	1	0	1	1	2.00	2	0	2	2	0	1
69	25.2	147	83	186	61	125	14.4	6	62	1	1	0	0	0	1	1	2.00	2	1	2	2	0	1
71	25.0	130	70	158	59	99	11.5	5	75	2	2	1	1	0	1	1	2.00	1	0	2	2	0	1
71	18.6	105	54	154	62	92	12.3	5	60	2	2	1	1	1	1	1	2.00	2	1	2	2	0	1
71	28.8	178	90	191	64	127	12.0	5	79	2	2	1	0	0	1	1	2.00	2	0	2	2	0	1
70	21.1	135	67	165	45	120	12.6	5	72	2	2	1	1	1	1	1	2.00	2	0	2	2	0	1
70	22.4	134	74	181	42	139	12.5	5	72	2	2	1	1	1	1	1	2.00	1	0	2	2	0	1
70	24.9	138	73	155	52	103	13.7	6	75	2	2	1	1	0	1	1	2.00	2	0	2	2	0	1
79	21.6	144	78	180	36	144	11.6	5	67	2	3	1	1	0	1	0	1.00	1	1	2	2	0	1
79	26.1	147	72	137	45	92	11.9	6	67	2	3	1	1	0	1	1	1.00	1	0	2	2	0	1
79	21.0	154	84	223	51	172	13.8	6	67	2	3	1	1	0	1	0	1.00	2	0	2	2	0	1
78	26.4	139	68	226	71	155	11.8	6	68	2	3	1	1	1	1	0	1.00	2	0	2	2	0	1
78	18.1	120	83	154	56	98	12.3	5	68	2	3	1	1	1	1	1	2.00	1	0	2	2	0	1

78	21.3	125	75	217	75	142	12.9	5	58	2	3	1	1	1	1	1	2.00	2	0	2	2	0	1
77	21.1	144	80	193	46	147	13.4	5	76	2	3	1	1	0	1	1	1.00	2	0	2	2	0	1
77	25.3	119	79	190	67	123	13.9	6	68	2	3	1	1	1	1	0	1.00	1	0	2	2	0	1
77	17.3	93	52	156	67	89	12.1	5	82	2	3	1	1	1	1	1	2.00	2	1	2	2	0	1
72	23.1	136	85	237	62	175	13.6	5	73	1	2	1	1	1	1	0	2.00	1	0	2	2	0	1
72	27.0	142	80	179	45	134	15.3	6	77	1	2	0	0	0	0	1	2.00	2	0	2	2	0	1
72	23.7	142	86	180	50	130	14.8	5	73	1	2	1	1	0	1	1	2.00	2	1	2	2	0	1
71	22.4	144	83	206	50	156	11.9	5	75	2	2	1	1	0	1	1	2.00	1	0	2	2	0	1
71	18.0	137	74	211	73	138	13.4	5	75	2	2	1	1	1	1	1	2.00	2	1	2	2	0	1
71	15.9	118	69	208	93	115	12.7	6	71	2	2	1	1	1	1	1	2.00	2	0	2	2	0	1
74	20.2	131	85	215	81	134	13.8	6	76	1	2	1	0	1	1	1	2.00	2	1	2	2	0	1
74	24.6	125	72	159	36	123	13.9	6	72	1	2	1	1	0	1	0	2.00	2	1	2	2	0	1
74	21.2	123	81	235	120	115	15.5	6	76	1	2	0	0	0	1	0	2.00	2	0	2	2	0	1
76	27.4	138	79	291	53	238	15.0	5	69	2	3	1	1	1	1	0	1.00	2	1	2	2	0	1
76	28.1	132	75	257	71	186	12.6	5	69	2	3	1	1	1	1	0	1.00	2	1	2	2	0	1
76	19.7	145	76	234	100	134	13.7	6	58	2	3	1	1	0	0	0	2.00	1	0	2	2	0	1
70	23.8	162	84	185	34	151	14.6	5	74	1	2	1	1	0	1	0	2.00	2	1	2	2	0	1
70	25.8	106	71	195	36	159	14.9	5	74	1	2	1	1	1	1	0	2.00	2	0	2	2	0	1
70	24.0	117	72	224	59	165	15.5	5	74	1	2	1	0	0	1	0	2.00	2	1	2	2	0	1
85	19.0	143	66	226	56	170	10.6	6	68	2	4	1	1	0	1	0	1.00	1	0	2	2	0	1
85	24.2	103	56	164	44	120	11.8	6	64	2	4	1	1	1	1	1	1.00	1	0	2	2	0	1
85	28.8	140	76	232	64	168	13.5	6	48	2	4	1	1	0	1	0	1.00	1	0	2	2	0	1
73	24.9	141	74	171	38	133	12.9	6	69	1	2	0	0	0	1	0	2.00	2	0	2	2	0	1
73	21.2	151	91	137	63	74	15.0	6	69	1	2	1	0	0	1	1	1.00	2	0	2	2	0	1
73	29.3	153	77	199	43	156	15.1	6	69	1	2	1	1	0	0	1	2.00	2	0	2	2	0	1
75	19.1	103	68	207	57	150	12.0	4	68	1	3	1	0	1	1	1	2.00	2	0	2	2	0	1
75	24.3	146	98	212	70	142	15.1	6	71	1	3	1	1	0	1	1	2.00	2	0	2	2	0	1
75	20.3	105	71	161	52	109	15.4	5	68	1	3	1	1	1	1	1	1.00	2	0	2	2	0	1
69	23.6	161	89	211	55	156	13.6	5	81	2	1	1	1	0	1	1	2.00	2	0	2	2	0	1
69	21.3	120	81	176	54	122	12.4	5	61	2	1	1	1	0	1	1	2.00	2	0	2	2	0	1
69	28.6	122	74	186	52	134	13.8	5	81	2	1	1	1	1	1	1	2.00	2	0	2	2	0	1
71	26.3	121	74	231	46	185	15.9	6	70	1	2	0	1	1	1	0	2.00	2	0	2	2	0	1
71	26.8	118	68	194	62	132	14.5	6	77	1	2	1	1	1	1	1	2.00	2	1	2	2	0	1
71	20.7	139	87	232	58	174	14.4	5	73	1	2	1	1	1	1	0	2.00	2	1	2	2	0	1
69	25.0	146	80	244	55	189	14.9	5	72	2	1	1	1	0	1	0	2.00	2	0	2	2	0	1
69	20.5	133	75	119	48	71	10.8	3	87	2	1	1	1	1	1	1	1.00	1	0	2	2	0	1
69	25.2	137	87	205	65	140	14.4	5	72	2	1	1	1	1	1	0	1.00	2	0	2	2	0	1
75	23.0	91	61	230	65	165	13.3	6	73	2	3	1	1	1	1	0	1.00	1	0	2	2	0	1
75	22.3	125	74	200	57	143	11.2	6	83	2	3	1	1	1	1	1	2.00	2	0	2	2	0	1
75	17.6	124	63	227	65	162	12.1	5	73	2	3	1	1	1	1	0	1.00	1	1	2	2	0	1
80	21.7	113	51	157	46	111	13.6	6	51	1	4	1	0	0	1	1	2.00	2	0	2	2	0	1
80	24.1	136	84	189	44	145	15.4	5	69	1	4	1	1	1	1	1	2.00	2	0	2	2	0	1
80	22.4	111	61	159	46	113	14.2	5	65	1	4	0	0	1	1	1	1.00	2	0	2	2	0	1
81	26.0	126	75	200	50	150	15.9	6	77	1	4	1	1	1	1	1	2.00	2	0	2	2	0	1
81	26.2	122	69	158	64	94	12.3	5	65	1	4	1	1	1	1	1	2.00	2	1	2	2	0	1
81	22.1	140	70	186	75	111	12.5	5	68	1	4	1	1	0	1	1	2.00	2	0	2	2	0	1
71	20.2	150	83	178	43	135	14.4	5	70	1	2	0	0	0	1	1	2.00	2	1	2	2	0	1
71	22.8	150	85	226	48	178	14.6	6	77	1	2	1	0	0	1	0	2.00	2	1	2	2	0	1
71	26.0	126	73	255	44	211	15.2	5	73	1	2	1	1	0	1	0	2.00	2	0	2	2	0	1
84	20.0	92	54	122	47	75	10.8	6	70	1	4	1	1	1	1	1	2.00	1	0	2	2	0	1
83	21.5	133	77	213	44	169	17.0	6	67	1	4	1	1	1	1	1	2.00	1	0	2	2	0	1

83	17.1	96	52	146	54	92	11.9	5	56	1	4	1	1	1	1	1	2.00	2	0	2	2	0	1
77	23.5	149	77	171	66	105	13.8	5	76	2	3	1	1	0	1	1	2.00	1	0	2	2	0	1
77	18.4	130	72	246	70	176	13.3	5	58	2	3	1	1	1	1	0	2.00	2	0	2	2	0	1
77	29.3	138	87	209	43	166	14.0	6	68	2	3	1	1	1	1	1	2.00	2	0	2	2	0	1
78	28.3	149	82	175	53	122	16.5	7	70	1	3	1	1	0	0	1	2.00	2	0	2	2	0	1
78	24.5	118	74	217	40	177	14.8	5	52	1	3	0	1	1	1	1	1.00	2	0	2	2	0	1
78	27.2	152	86	172	40	132	12.5	6	70	1	3	1	1	0	1	1	2.00	2	0	2	2	0	1
76	26.2	115	72	271	57	214	13.7	6	58	2	3	1	1	0	1	0	1.00	2	0	2	2	0	1
76	25.1	151	80	209	57	152	12.2	5	69	2	3	1	1	0	1	1	1.00	2	0	2	2	0	1
76	24.2	111	71	159	70	89	13.6	5	72	2	3	1	1	1	1	1	2.00	1	0	2	2	0	1
74	25.4	148	87	135	42	93	15.6	5	68	1	2	1	1	0	1	1	2.00	2	0	2	2	0	1
74	26.6	132	77	193	44	149	15.4	6	68	1	2	1	1	0	1	1	2.00	2	1	2	2	0	1
74	22.7	168	70	230	54	176	13.7	6	72	1	2	0	1	0	1	0	2.00	1	1	2	2	0	1
71	21.3	166	97	218	74	144	14.9	5	73	1	2	1	1	0	1	1	2.00	2	1	2	2	0	1
71	25.6	97	58	149	48	101	14.6	5	73	1	2	1	1	1	1	1	2.00	2	1	2	2	0	1
71	21.5	133	75	182	60	122	14.1	5	73	1	2	1	1	1	1	1	2.00	2	0	2	2	0	1
80	22.7	91	54	181	46	135	11.3	6	70	2	4	1	1	1	1	1	1.00	1	0	2	2	0	1
80	23.8	141	84	256	70	186	12.8	5	67	2	4	1	1	0	1	0	1.00	1	0	2	2	0	1
80	26.6	136	70	211	54	157	13.5	6	49	2	4	1	1	0	1	1	2.00	2	0	2	2	0	1
82	20.8	138	83	156	55	101	12.1	6	71	1	4	1	1	1	1	1	2.00	1	0	2	2	0	1
82	21.4	134	72	185	58	127	13.6	5	68	1	4	0	0	1	1	1	2.00	2	1	2	2	0	1
82	24.7	112	65	140	44	96	11.5	6	51	1	4	1	1	1	1	1	1.00	2	0	2	2	0	1
76	20.2	145	86	179	65	114	14.3	6	75	1	3	1	1	0	1	1	2.00	2	1	2	2	0	1
76	23.5	103	57	182	44	138	13.0	5	67	1	3	1	1	1	1	1	2.00	2	0	2	2	0	1
76	22.1	155	84	182	41	141	14.4	5	59	1	3	1	1	0	1	1	2.00	2	1	2	2	0	1
80	22.0	125	67	200	78	122	12.7	6	75	2	4	1	1	0	1	0	2.00	1	0	2	2	0	1
80	30.2	134	76	175	35	140	13.9	6	75	2	4	1	1	0	1	0	1.00	1	0	2	2	0	1
80	28.0	135	72	174	46	128	12.1	9	75	2	4	1	1	0	0	1	1.00	1	0	2	2	0	1
79	23.3	126	77	241	77	164	13.2	5	71	2	3	1	1	1	1	0	1.00	2	0	2	2	0	1
79	23.9	147	83	176	57	119	13.7	5	71	2	3	1	1	0	1	1	1.00	1	0	2	2	0	1
79	23.0	153	84	213	64	149	13.9	5	71	2	3	1	1	0	1	1	2.00	1	1	2	2	0	1
