# Supplementary material for: Low educational level increases functional disability risk subsequent to heart failure in Japan: On behalf of the Iwate KENCO study group
Source: PLoS One. 2021 Jun 8;16(6):e0253017. doi: 10.1371/journal.pone.0253017 (PMC8186788; doi:10.1371/journal.pone.0253017)
Supplement: S3 Table — (PDF) [file pone.0253017.s003.pdf]

S3 Table. Explanation of variables

Variable label	Variables	Value label
Age (years)	age	
Body mass index (kg/m^2)	bmi	
Systolic blood pressure (mmHg)	sbp	
Diastolic blood pressure (mmHg)	dbp	
Total cholesterol (mg/dl)	tc	
High-density lipoprotein cholesterol (mg/dl)	hdlc	
Non high-density lipoprotein cholesterol (mg/dl)	nonHDLC	
blood hemoglobin (g/dl)	hb	
Glycemic hemoglobin; HbA1c (NGSP) (%)	Hba1c_N	
eGFR (mL/min/1.73 m^2)	egfr_epi	
Sex	sex	1, males; 2, females
Age group	age_n	1, 65-69 years old, 2, 70-74 years old, 3, 75-79 years old, 4, 80 years or older
Current smoker	TBC_SITU_c	0, current smoker; 1, non-smoker
Current drinker	ALC_SITU_c	0, regular drinker; 1, non regular drinker
Hypertension	t1htt_in	0, hypertension; 1, non hypertension
Diabetes mellitus	dm_ni	0, diabetes mellitus; 1, non diabetes mellitus
Dyslipidemia	t1lipid_o	0, dyslipidemia; 1, non dyslipidemia
Marrital status	Q_MARR_s	1, unmarried; 2, married
Educational years	Q_school_t	1, low educational years (< 7 years); 2 high educational years (≥ 7 years)
Job status	Q05_s	0, unemployed; 1, employed
Long-term care after heart failure	Bunrui	1, No long-term care after HF; 2, Long-term care after heart failure
Heart failure	y_moku2	1, heart failure case; 2, no heart failure
Heart failure	y_moku	0, no heart failure; 1, heart failure
Subjects without HF cases within two years from the baseline su	filter_Apr0220x	0, excluded subjects; 1, included subjects
